# Evaluation of Anti-Tumor Effects of Whole-Body Low-Dose Irradiation in Metastatic Mouse Models

**DOI:** 10.3390/cancers12051126

**Published:** 2020-04-30

**Authors:** Kyung-Hee Song, Seung-Youn Jung, Jeong-In Park, Jiyeon Ahn, Jong-Kuk Park, Sang-Gu Hwang, Eun-Ho Kim, Seon Young Nam, Seungwoo Park, Hunjoo Ha, Jie-Young Song

**Affiliations:** 1 Division of Radiation Biomedical Research, Korea Institute of Radiological & Medical Sciences, Seoul 01812, Korea; songkh@kirams.re.kr (K.-H.S.); seungyoun@kirams.re.kr (S.-Y.J.); jipark@kirams.re.kr (J.-I.P.); ahnjy@kirams.re.kr (J.A.); jkpark@kirams.re.kr (J.-K.P.); sgh63@kirams.re.kr (S.-G.H.); eh140148@cu.ac.kr (E.-H.K.); 2 Graduate School of Pharmaceutical Sciences, College of Pharmacy, Ewha Womans University, Seoul 03760, Korea; 3 Department of Biochemistry, School of Medicine, Daegu Catholic University, Daegu 42472, Korea; 4 Low-Dose Radiation Research Team, Radiation Health Institute, Korea Hydro & Nuclear Power Co., Ltd., Seoul 01450, Korea; namsy6660@khnp.co.kr; 5 Division of Medical Radiation Equipment, Korea Institute of Radiological & Medical Sciences, Seoul 01812, Korea; swpark@kirams.re.kr

**Keywords:** irradiation, systemic immune response, epithelial–mesenchymal transition, metastasis, abscopal effect

## Abstract

Low-dose irradiation (LDI) has recently been shown to have various beneficial effects on human health, such as on cellular metabolic activities, DNA repair, antioxidant activity, homeostasis potency, and immune activation. Although studies on the immunogenic effects of LDI are rapidly accumulating, clinical trials for cancer treatment are considered premature owing to the lack of available preclinical results and protocols. Here, we aim to investigate anti-tumor and anti-metastatic effects of whole-body LDI in several tumor-bearing mouse models. Mice were exposed to single or fractionated whole-body LDI prior to tumor transplantation, and tumor growth and metastatic potential were determined, along with analysis of immune cell populations and expression of epithelial–mesenchymal transition (EMT) markers. Whole-body fractionated-LDI decreased tumor development and lung metastasis not only by infiltration of CD4^+^, CD8^+^ T-cells, and dendritic cells (DCs) but also by attenuating EMT. Moreover, a combination of whole-body LDI with localized high-dose radiation therapy reduced the non-irradiated abscopal tumor growth and increased infiltration of effector T cells and DCs. Therefore, whole-body LDI in combination with high-dose radiation therapy could be a potential therapeutic strategy for treating cancer.

## 1. Introduction

According to many studies on the biological effects of low-dose irradiation (LDI), defined as doses less than 100 mGy, LDI has been shown to induce beneficial effects from bacteria to humans through diverse cellular changes, including adaptive responses, bystander effects, genomic instability, and coordinated response [1,2,3,4]. These observations cannot be explained by the linear-no-threshold hypothesis, which is adopted to establish radiation protection guidelines to define how health risks increase linearly based on the radiation dose without a threshold [5]. This hypothesis suggests that no matter how low the radiation dose is, it is harmful and increases the risk to health. In reality, humans are continuously exposed to naturally occurring background radiation from cosmic and terrestrial radiation and manmade sources, averaging an annual dose of approximately 2.4 millisievert (mSv) and ranging from 1–13 mSv, but which can exceed 50 mSv [6,7]. Despite the great interest and accumulated evidence of the effects of LDI, there are often contradictory results depending on the radiation dose, dose rate, irradiation schedule, experimental method, and the subjects used in the study. 

Recently, given preclinical studies suggesting that LDI can activate immune responses and enhance the efficacy of cancer therapy, several studies have explored the combined effects of LDI and immunotherapy (IT) or radiation therapy (RT) [3,8]. The immune system is a powerful cellular network that provides surveillance to prevent the development and progression of malignancy. Numerous studies have indicated that the effect of RT on tumor microenvironment (TME) may play an important role in therapeutic outcomes owing to the differential activation of survival and death program in diverse cell components by multiple biological processes. The therapeutic efficacy of RT involves the elimination of irradiated cancer cells, as well as the activation of tumor-targeting immune response that operates locally and systemically to control metastatic lesions [9]. The latter phenomenon, known as the abscopal effect, was first described by Mole in 1953 and refers to immune-mediated responses of distal lesions that are away from the irradiation site [10,11,12]. RT to local tumors can generate and release tumor-associated neoantigens, resulting in the elicitation of antigen-specific T cells and a significant reduction in systemic tumor burden in a CD8^+^ T-cell-dependent fashion [13,14,15,16]. In support of this observation, exogenous administration of chemokines following local RT can inhibit tumor growth at distal site, and stimulate infiltration of CD8^+^ T-, CD4^+^ T cells, and NK cells [17]. In early studies in mouse models of metastatic breast cancers, RT combined with an inhibitor of cytotoxic T-lymphocyte associated protein-4 (CTLA-4), a representative negative immune checkpoint, demonstrated a significant survival benefit compared to RT alone. Subsequent human studies with CTLA-4 and programed cell death-1 (PD-1) blockade have shown improved overall survival in patients with metastatic melanoma, as well as non-small cell lung cancer, breast, and renal cell carcinoma [18,19,20,21]. Thus, enhancing the immune system can retard the progression of cancer cells and prevent escape from immune surveillance, providing a potential strategy for long-term anti-tumor response [22].

Our previous study demonstrated that LDI can modulate immunological responses via the differential activation of T-, B-, NK cells, dendritic cells (DC), and macrophages, according to the dosing method and time [23]. In addition, LDI reduces lethality in septic mice by enhancing bacterial clearance with macrophage activation [24]. Likewise, one report has shown that LDI could upregulate several genes to release immune-related cytokines that modulate the activity of CD4+ T cells [25]. LDI enhances effector T-cell function and prolongs mouse survival, while selectively decreasing the infiltration of immunosuppressive regulatory T cells (Treg) in tumor tissues [26]. Recently, it was shown that LDI attenuates epithelial–mesenchymal transition (EMT) and stemness in breast cancer cells, leading to impaired cancer metastasis [27,28]. These findings suggest that the anti-tumor effect of LDI could be attributed to suppression of tumor progression by EMT, as well as activation of immune cells. However, no study has demonstrated if whole-body fractionated LDI possesses anti-tumor and anti-metastatic activity in in vivo mouse models. Although several in vitro studies have demonstrated anti-tumor and anti-metastatic effects of whole-body LDI, this study aimed to determine whether whole-body LDI exerts the same effects in vivo, and provides a promising avenue for cancer treatment.

## 2. Results

### 2.1. LDI Inhibits Experimental Lung Metastasis of B16F10 Melanoma

**Figure 1:**
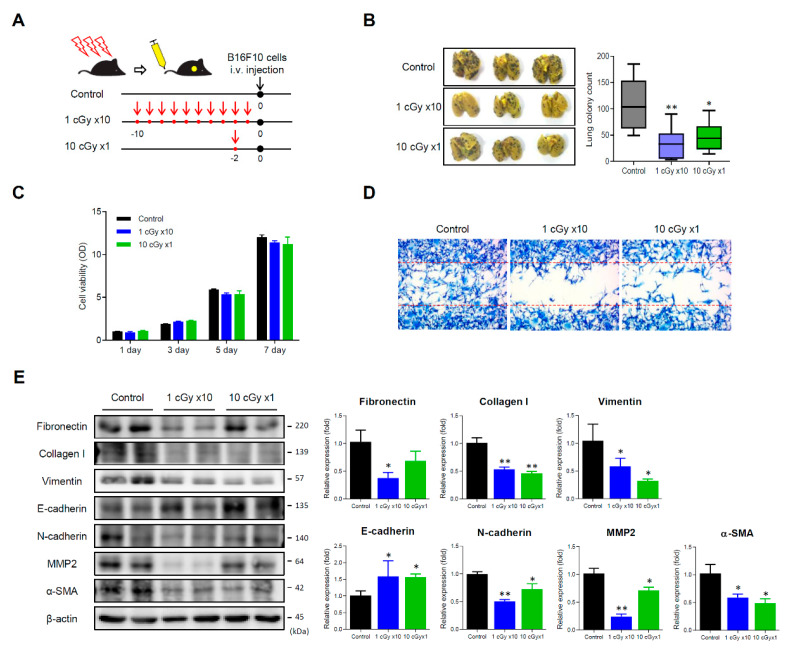
Low dose irradiation (LDI) inhibits the lung metastasis in B16F10 melanoma-bearing mice. (**A**) Experimental scheme of LDI exposure to mice. Mice were either irradiated with 10 cGy at once or 1 cGy 10 times (accumulative dose of 10 cGy), and then B16F10 (1 × 10^6^ cells) were intravenously injected into tail vein. (**B**) Photograph of lungs after soaking in Bouin’s solution. The number of metastatic foci were counted and calculated in each group. Data represent means ± SEM of 6–8 mice experiment. * *p* < 0.05, ** *p* < 0.01 vs. control. (**C**) Cell viability isolated from the lungs was determined using a 3-[4,5-Dimethylthiazol-2-yl]-2,5-diphenyltetrazolium bromide (MTT) assay. See Section 4.6 for details (**D**) The effect of LDI on cell migration was measured via wound scratch assay. (**E**) The expression of epithelial–mesenchymal transition (EMT)-related proteins in the lungs was examined by Western blotting. Data represent means ± SEM of three independent experiments. * *p* < 0.05, ** *p* < 0.01 vs. control. Detailed information about western blot can be found at Figure S1.

Given the previous findings on the effect of LDI on anti-tumor and immunomodulatory activity, we initially examined the effect of whole-body LDI in experimental models of lung metastasis. We used the metastatic melanoma cell line B16F10, which was selected in vivo on the basis of its high capacity to colonize in lungs of mice, by intravenous injection into the lateral tail vein [29]. After exposing mice to whole-body irradiation (Figure 1A), both the number of metastatic tumor foci in the LDI-treated mice were significantly decreased compared with that in control mice (Figure 1B). To examine whether whole-body LDI alters the characteristics of tumor cells, B16F10 cells from lungs were isolated, and the proliferative activity was measured. As the proliferative activity of isolated B16 cells from whole-body LDI-exposed mice was not changed compared with that from control mice (Figure 1C), we performed wound-healing assay to measure metastatic potential. The isolated cells from whole-body LDI-treated mice showed a significant reduction in cell migration to the wound, and this effect was more pronounced in the fractionated-LDI (Figure 1D). In addition, isolated cells from both LDI-treated mice markedly diminished the expression of mesenchymal markers such as fibronectin, collagen type 1, vimentin, N-cadherin, α-SMA, and MMP2. However, E-cadherin, an epithelial marker, was reversely increased in the LDI-treated mice (Figure 1E). 

### 2.2. LDI Suppresses Spontaneous Metastasis of 4T1 Cells

**Figure 2:**
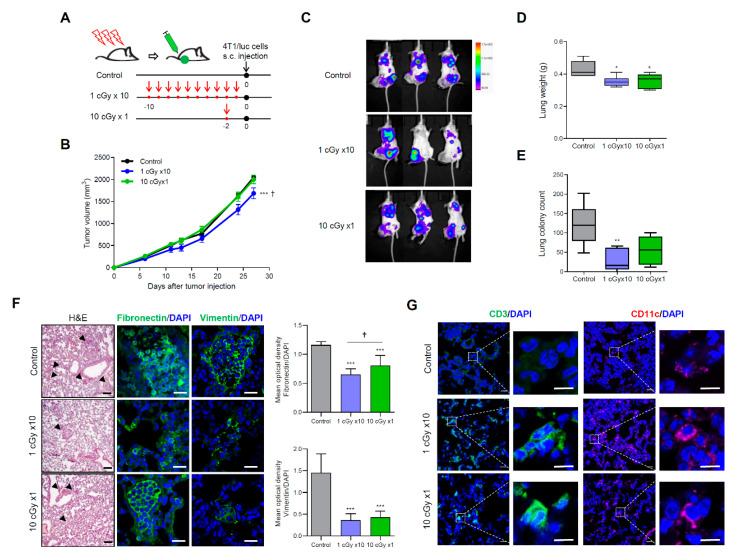
LDI reduces the metastatic potential of 4T1 cell-bearing mice. (**A**) Experimental schematic of LDI exposure to mice. Mice were either irradiated with 10 cGy at once or 1 cGy for 10 times (accumulative dose of 10 cGy), and then 4T1/luc (1 × 10^6^ cells) were subcutaneously injected into the right thigh. (**B**) Tumor growth curve are shown after 4T1/luc inoculation. (**C**) Bioluminescent intensity of representative mice monitored at week 34 after injection of 4T1/luc cells. (**D**) Lungs were weighed and reported. (**E**) The number of metastatic foci were counted and calculated in each group. Data represent means ± SEM of 6–7 mice per experiment. * *p* < 0.05, ** *p* < 0.01, *** *p* < 0.001 vs. control, † *p* < 0.05 vs. 10 cGy × 1. (F) Lung tissue sections of mice were stained with hematoxylin and eosin staining (magnification ×100, scale bar 100 μm) and fibronectin and vimentin by immunofluorescence staining (magnification ×400, scale bar 20 μm). The mean optical density of the fluorescence signals from each protein were quantified and normalized using DAPI. Data represent means ± SEM of 8 images. *** *p* < 0.001 vs. control, † *p* < 0.05 vs. 10 cGy × 1. (G) The recruitment of CD3+ lymphocytes and CD11c+ dendritic cells in lung tissues were stained using immunofluorescence staining. Cell nuclei are stained blue by DAPI. Magnification ×400, crop image scale bar 10 μm.

To confirm the effect of LDI in spontaneous metastatic models, mice were subcutaneously injected with highly metastatic 4T1 cells transduced with firefly luciferase (4T1/luc) (Figure 2A). The tumor growth of 4T1/luc cells in single LDI-treated mice was not different from non-irradiated control mice, whereas tumor growth significantly decreased in the fractionated LDI-exposed group (Figure 2B). The measurement of luciferase activity indicated that fractionated LDI more significantly inhibits lung metastasis than a single dose of LDI (Figure 2C). After sacrifice, the weight and metastatic colonies of lungs were measured. The weight of lungs and the number of metastatic foci were effectively decreased in fractionated and single LDI-treated mice than in non-irradiated control mice (Figure 2D,E). Of note, fractionated LDI more significantly decreased the lung metastatic nodules (27.7 ± 11.5) than control treatment (120.0 ± 25.6) or single-dose LDI (54.4 ± 14.4). Moreover, hematoxylin and eosin staining showed a reduction in the tumor mass in the lungs of LDI-treated mice with significant suppression of fibronectin and vimentin expression (Figure 2F). When assessing infiltration of immune cells in the lungs using immunofluorescence staining, the accumulation of CD3 (T-lymphocytes) and CD11c (dendritic cells) was observed in LDI-treated mice (Figure 2G). 

### 2.3. LDI Induces Abscopal Effects

**Figure 3:**
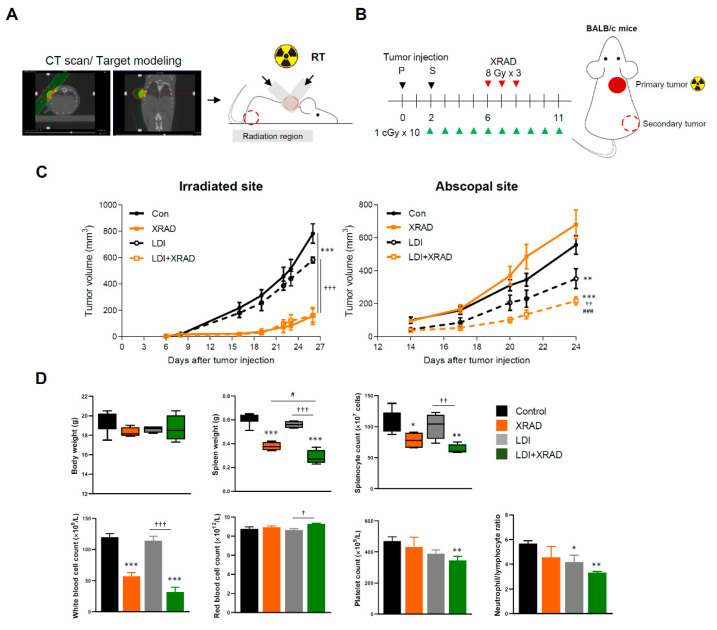
LDI enhances the abscopal effect combined with high-dose RT. (**A**) Schematic representation of the X-RAD SmART (small animal image-guided radiation therapy research system; Precision X-ray Inc., North Branford, CT, USA) irradiator setup. Acquiring cone beam computed tomography (CBCT) scans to precisely place the isocenter to the treatment location and visualize the internal structure of mice. X-RAD SmART Plan to calculate dose, provides complex beam planning. (**B**) Experimental scheme of tumor inoculation and irradiation interval to mice as described in the Methods section. (**C**) Tumor growth was monitored with irradiated and abscopal tumors in each group. (**D**) Body and spleen weight were measured at the end of the experiment. Splenocyte number was determined using a Trypan blue exclusion assay. Whole blood was drawn by retro-orbital puncture and analyzed using a VETSCAN HM5 hematology analyzer (Abaxis, Union City, CA, USA). Data represent means ± SEM of 6-8 mice per experiment. * *p* < 0.05, ** *p* < 0.01, *** *p* < 0.001 vs. control, † *p* < 0.05, †† *p* < 0.01, ††† *p* < 0.001 vs. LDI, # *p* < 0.05, ### *p* < 0.001 vs. XRAD.

Given that the fractionated LDI had higher anti-metastatic activity than a single dose of LDI, we further investigated whether fractionated LDI may be sufficient to stimulate the systemic immune response in combination with high-dose RT (8 Gy for three times) treatment on local tumors. BALB/c mice were inoculated 4T1 cells at two separate sites, in the back (primary tumors) and in the right thigh (secondary tumor) (Figure 3A). The primary tumor received high-dose RT simultaneously with whole-body fractionated LDI, whereas the secondary tumor was not irradiated (Figure 3B). The high-dose RT showed a significant growth delay of primary tumor, but had no effect on secondary tumor growth. Interestingly, whole-body fractionated LDI alone slightly suppressed the primary tumor volume without statistical significance. However, fractionated LDI by itself not only led to significant growth delay of the secondary tumor, but also resulted in a more pronounced anti-tumor effect in combination with high-dose RT (Figure 3C). There was no difference in body weight between all experimental groups, but spleen mass and splenocyte number were reduced by XRAD treatment. In addition, the results of complete blood count analysis revealed significant changes in white blood cells, platelets, and neutrophils-to-lymphocytes ratio (NLR) in LDI + XRAD (high-dose RT) group compared with those in other groups (Figure 3D). 

### 2.4. LDI Synergizes with RT Via EMT Inhibition

**Figure 4:**
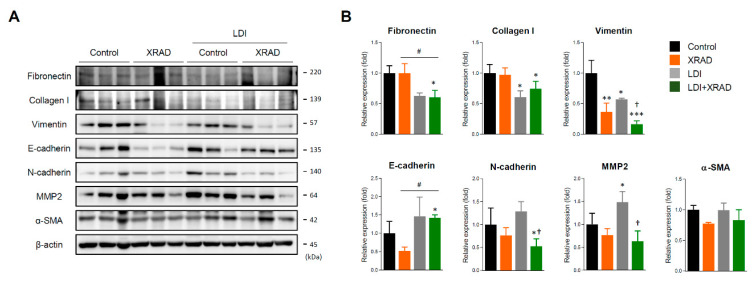
LDI synergistically inhibits EMT proteins combined with high-dose RT. (**A**) The expression of EMT proteins such as fibronectin, collagen I, vimentin, E-cadherin, N-cadherin, MMP2, and α-SMA in primary tumor were detected by Western blot. (**B**) The relative expression of proteins was quantified by densitometry using Image J software and normalized by the levels of β-actin. Data represent means ± SEM of three independent experiments. * *p* < 0.05, ** *p* < 0.01, *** *p* < 0.001 vs. control, † *p* < 0.05 vs. LDI, # *p* < 0.05 vs. XRAD. Detailed information about western blot can be found at Figure S2.

To investigate that whole-body LDI causes some changes in TME and induces systemic immunity to inhibit tumor growth and micrometastases, we determined the expression of EMT molecules in primary tumors. LDI alone significantly decreased the expression levels of fibrotic and mesenchymal molecules such as fibronectin, collagen I, and vimentin, and showed similar expression levels in comparison to high-dose RT. In addition, high-dose RT combined with LDI suppressed the expression of EMT molecules such as fibronectin, vimentin, N-cadherin, and MMP2 to a greater extent than high-dose RT or LDI treatment alone. On the contrary, expression of the E-cadherin was significantly increased only upon treatment with high-dose RT combined with LDI (Figure 4). 

### 2.5. LDI Induces the Abscopal Effect Via Stimulation of Immune Response

**Figure 5:**
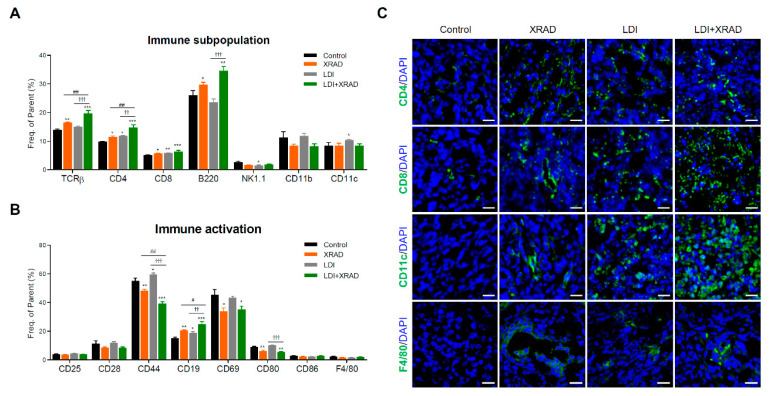
LDI significantly increases immune subpopulation and activation in splenocytes. (**A**) Subpopulation of splenic immune cells and (**B**) activation markers in the spleen were labeled using fluorescence-labeled monoclonal antibodies and examined by flow cytometry. Data represent means ± SEM of 6-8 mice per experiment. * *p* < 0.05, ** *p* < 0.01, *** *p* < 0.001 vs. control, †† *p* < 0.01, ††† *p* < 0.001 vs. LDI, # *p* < 0.05, ## *p* < 0.01 vs. XRAD. (**C**) Representative confocal sections showing immune cell recruitment including CD4, CD8, CD11c and F4/80 I then secondary tumor site were detected by immunofluorescence staining. Cell nuclei are stained blue by DAPI. Scale bar, 20 μm.

To examine LDI-mediated systemic immunity, subpopulations of splenocytes were analyzed by flow cytometry. The high-dose RT caused an increase in T cell (TCRβ, CD4, and CD8) and B cell (B220) populations. In contrast, RT resulted in a mild decrease in the number of NK cells (NK1.1) and macrophages (CD11b), although this finding was not statistically significant. The population of CD4^+^ and CD8^+^ T cells and DCs (CD11c) in the splenocytes of LDI-exposed mice was noticeably increased compared with that in splenocytes of control mice. The combination of high-dose RT and LDI more increased the number of T and B cells in the spleen (Figure 5A). Along with the number of immune cells, the activation markers of splenic leucocytes were also examined to determine whether increased immune cells actually retain their function. Interestingly, in contrast to the increased number of T cells by high-dose RT with or without LDI, the levels of CD25^+^, CD28^+^, and CD44^+^ T cells were decreased. However, as the number of B cells increased, the frequency of active CD19^+^ B cells also increased (Figure 5B). We further investigated the intratumoral immune cell infiltration in secondary tumor sites using immunofluorescence staining. LDI strongly increased the infiltration of CD4^+^, CD8^+^ T cells, CD11c^+^ dendritic cells, and F4/80 macrophages, and this effect increased upon treatment with the combination of LDI and high-dose RT (Figure 5C). Although high-dose RT had no potential for abscopal effect in secondary tumors, the number of CD4^+^ and CD8^+^ T cells, DCs, and macrophages was slightly increased compared with that after control treatment, similar to the subpopulations of splenocytes. 

### 2.6. Abscopal Effect of LDI in Immunodeficient Nude Mice

**Figure 6:**
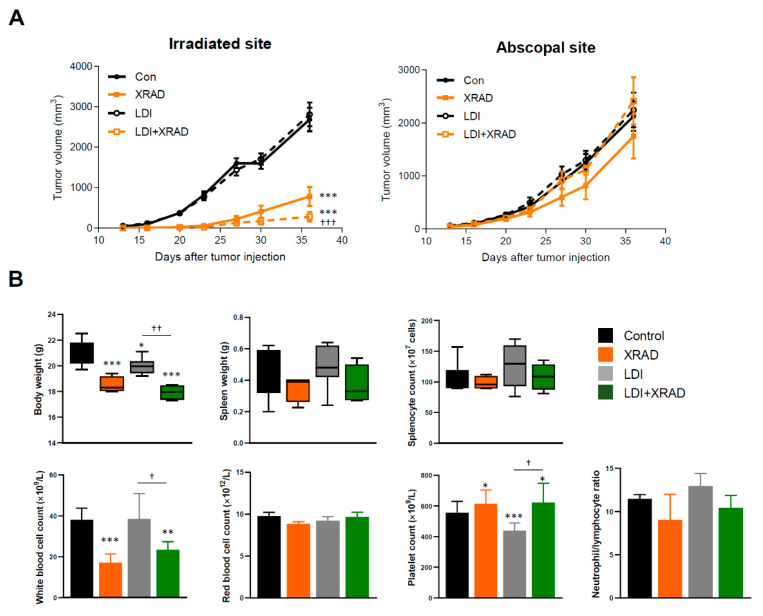
LDI-mediated abscopal response was not applied to immunodeficient mice. (**A**) Tumor growth was monitored with irradiated and abscopal tumors in each group. (**B**) Body and spleen weight were measured at the end of the experiment. Splenocyte number was determined using a Trypan blue exclusion assay. Whole blood was drawn by retro-orbital puncture and analyzed using a VETSCAN HM5 hematology analyzer. Data represent means ± SEM of 6–8 mice per experiment. * *p* < 0.05, ** *p* < 0.01, *** *p* < 0.001 vs. control, † *p* < 0.05, †† *p* < 0.01, ††† *p* < 0.001 vs. LDI.

To confirm the importance of immune response in LDI-mediated abscopal effects, immunodeficient BALB/c nude mice were employed for the above experimental conditions. As shown in Figure 3C, LDI treatment in immunocompetent mice inhibited secondary tumor growth, demonstrating that LDI contributes to the abscopal effect for suppression of tumor growth. However, these effects were not observed in immunodeficient athymic mice that have defects in the differentiation and maturation of T cells (Figure 6A). In contrast to the results in Figure 3D, body weight was reduced in all treatment groups compared to the control group, but no significant change was observed in spleen mass and splenocyte number. Moreover, compared with immunocompetent mice, reduced platelet count and NLR in the LDI + XRAD group were restored to control levels in immunodeficient mice (Figure 6B). 

## 3. Discussion

Many researchers agree that, unlike high-dose radiation, cellular responses to absorbed LDI are not easily predictable, as it is difficult to accurately measure the energy distribution of low-dose exposed tissues. In addition, the biological effects of LDI are so weak and short in duration that there are limits in detection by currently common techniques. However, accumulating evidence indicates that LDI appears to be related to hormesis, bystander effects, and adaptive responses [30,31,32,33] in several disease conditions such as Alzheimer’s disease [34], rheumatoid arthritis [35], systemic lupus erythematosus [36], multiple sclerosis [37] and type-1 diabetes [38]. Moreover, the therapeutic effect of LDI has been shown to kill tumor cells [27,39,40,41]. Nevertheless, the biological impact of LDI varies in certain circumstances, and because the exact mechanisms are still largely unknown, various scientific studies are extensively underway. 

In the present study, we aim to determine the anti-tumor and anti-metastatic effects of whole-body LDI in in vivo tumor-transplanted mouse models. Although several studies have shown that LDI suppressed the development of primary or metastatic tumors [42,43,44,45], further studies are needed to arrive at a unified conclusion, since different experimental designs and RT protocols have been used. Moreover, most studies have been performed using single-dose radiation; as a result, few data are available regarding the effect of fractioned low-dose radiation to tumor progression and metastasis. Our results indicated that whole-body LDI suppressed lung metastases in artificially and intravenously injected melanoma mouse models, accompanied with substantial downregulated invasion activity and decreased expression of EMT-related proteins (Figure 1). Numerous evidence suggests that EMT is associated with cell motility, invasiveness, metastasis, tumor stemness, and resistance to cancer therapy [46,47]. Similar results were observed in spontaneous metastatic model of 4T1-transplanted mice (Figure 2), and the fractionated radiation was more effective than single dose of radiation. Apart from these findings, we also investigated tumor growth by transplantation of in vitro LDI-treated 4T1 cells (Figure S3). Mice transplanted with single or fractionated LDI-treated cells showed significantly delayed tumor growth compared with non-irradiated cell injected mice, which was even more pronounced compared to the experimental results in Figure 1. Consistent with our results, Kaushik et al. showed that LDI decreased tumor progression by suppressing EMT and cancer stem-cell maintenance in breast cancer cell lines [27]. In addition, preclinical studies have shown that LDI converts tumor stroma to a more favorable environment via the regulation of cytokine production, tumor vasculature, and oxidative stress [8,48,49].

The relationship between immunity and metastatic cancer has been well studied and the generation of the pre-metastatic niches in the lungs is highly dependent on immunosuppression to block T cells, NK cells and monocytes from the presence of tumor cells to establish metastatic lesions [50,51,52,53,54]. Until recently, RT was considered to be immunosuppressive to cause direct localized normal cell death and apoptosis in immune cells, but accumulating evidence has proved that RT can activate the immune system and elicit systemic anti-tumor responses to regress metastatic cancer at distant sites. Specifically, LDI-mediated immunoactivation promoted the proliferation of splenic and thymic lymphocytes and increased cytokines release, and thereby the suppression of tumor growth [28]. According to our previous data, the pre-LDI prolonged the survival of septic mice by increasing the number of immune cells, as well as activating immunological functions to eradicate bacterial pathogens [24]. Moreover, single or fractionated LDI contribute to differential immune responses, such as Th1 type activation or Th2 shift in normal mice [23]. In the current study, we observed that whole-body LDI increased the populations of CD3^+^ T cell and CD11c^+^ DCs in metastasized lungs, indicating that LDI could prevent metastases not only by immune cell infiltration, but also by alterations in TME. However, the underlying mechanisms and key players involved in these functions remain to be clarified.

In agreement with the previous report that a single, local RT did not reproduce the abscopal effect consistently [55], high-dose RT alone did not inhibit the non-irradiated secondary tumor growth in the present study. X-RAD group rather showed a non-statistically significant increase in tumor growth at the abscopal site (*p* value = 0.1735 vs. control). Habets et al. showed that the administration of RT (8 Gy × 3) to the primary 67NR tumor prolonged survival and delayed the growth of both primary and secondary tumor, as compared with the control [56]. In contrast, Liu et al. demonstrated that RT (8 Gy × 3) led to a significant growth delay in the irradiated primary 4T1 tumor but did not inhibit secondary tumor growth [3]. Zhang X. et al. similarly reported that local irradiation with 3 × 9.18 Gy on primary melanoma tumor in two B16-CD133 in mice did not induce any abscopal effect [57]. It is expected that the overall occurrence rate of abscopal effects was relatively low due to the insufficiency of RT treatment alone to overcome the immunoresistance of malignant tumors [58]. Interestingly, fractionated whole-body LDI significantly delayed secondary tumor growth and enhanced anti-tumor effect in combination with high-dose RT (Figure 3). It may be assumed that consecutive LDI can modulate TME, induce immune cell infiltration, overcome the immunoresistance of malignant tumors, and trigger systemic anti-tumor effects, eventually resulting in the abscopal effect. Additionally, the optimal dosing and fractionation schemes of RT and predictive biomarkers of RI-induced immune priming for each cancer type remain a big challenge. Recently, Vanpouille-Box et al. demonstrated that ablative hypo-fractionated radiation (such as three rounds of 8 Gy) inhibited DNA exonuclease TREX1 and activated cGAS-STING axis, thereby leading to IFN-β production by tumor cells that stimulate immune activation in different colorectal carcinoma and breast cancer cell lines [59]. Furthermore, preclinical and clinical studies have demonstrated that the abscopal effect of RT distinctively appeared when combined with IT, such as the presence of anti-CTLA4 or PD-1 antibodies, and anti-tumor cytotoxic T cells play a major role in that effect [60]. Therefore, we employed the hypo-fractionated RT (three rounds of 8 Gy) in combination with consecutive LDI to manifest the systemic immunity and abscopal effect. We observed that decreased expressions of EMT- related proteins in the primary tumors (Figure 4), and accumulation of CD4^+^ and CD8^+^ T cells and other immune cells in the non-irradiated secondary tumors (Figure 5), as well as a significant inhibition of tumor growth. This result suggests that our treatment protocol is suitable for inducing sufficient immune activation to increase the number and the function of T cells in tumors. Similarly, Liu et al. revealed that a single pretreatment of LDI prior to hypo-fractionated RT can enhance systemic and tumor-specific immune response with significant CD8^+^ T cells and DCs infiltration in the non-irradiated tumor [3]. 

As expected, the reduction in primary and secondary tumor volume by fractionated LDI was not present in immunodeficient mice (Figure 6). In addition, the significant delay in secondary tumor growth induced by LDI with high-dose RT in immunocompetent mice was not observed in immunodeficient mice under the same experimental conditions, confirming that the anti-tumor and abscopal effect of LDI are attributed to the stimulation of immunity. In addition, the platelet level and NLR in immunodeficient mice was different from that in immunocompetent mice. Accumulating evidence demonstrated that elevated counts of blood platelets and neutrophils have been highly associated with tumor progression and metastasis, and NLR has been proposed as an attractive prognostic factor for survival in many types of cancers [61,62]. This result suggests that any factors suppressing the immune system could interfere with the generation of an abscopal response. Indeed, a number of limitations can hamper the immune-mediated abscopal effect in clinical data, such as the patient’s degree of myelosuppression, overall tumor burden, NLR, as well as the patient’s experience of RT or chemotherapy [63]. As escape of immune surveillance is a hallmark of cancer, immune cell depletion and immunosuppressive TME is commonly observed in most cancer patients, which reduces the efficacy of anticancer therapies and causes resistance and relapse [64,65,66]. Therefore, the immunogenic tumor environment and priming of immune responses by LDI may be a good approach in treating metastatic cancer and may help to improve response to combination therapies. Because this study has certain limitations, such as insufficient sample sizes and treatment protocols, lack of different mouse strains, and absence of blocking or rescue experiments on specific immune cells, further investigation is required to provide a clearer understanding of the biological effect of LDI. Moreover, since the risks, as well as the benefit of LDI to living organisms is still controversial, the cumulative or latent toxicity of fractionated LDI and clinical utility must be thoroughly evaluated.

This study, for the first time, demonstrated that whole-body fractionated LDI exhibits anti-tumor and anti-metastatic activity in vivo in three mouse models, via the alteration in immunosuppressive TME, further enhancing the high-dose RT-mediated anti-tumor responses. These findings emphasized the importance of the immune response in therapeutic strategies involving RT, and also demonstrated the potential of LDI-mediated immune activation in increasing the anti-tumor effect of RT. 

## 4. Materials and Methods

### 4.1. Cell Lines

Murine triple-negative breast cancer cell line 4T1, melanoma cell line B16F10, and human triple-negative breast cancer cell MDA-MB-231 were purchased from the American Type Culture Collection (Manassas, VA, USA). The cells were grown in RPMI-1640 or DMEM (Welgene, Gyeongsan-si, Gyengsangbuk-do, Korea) supplemented with 10% fetal bovine serum (Welgene), 100 IU/mL penicillin, and 100 μg/mL streptomycin (Invitrogen, Carlsbad, CA, USA) and maintained at 37 °C in a humidified atmosphere of 5% CO_2_. For appraisal of tumor burden in metastatic organs, 4T1 were transfected with firefly luciferase expressing vector using Lipofectamine 2000 (Thermo scientific, Waltham, MA, USA) and selected in the medium containing 200 μg/ml hygromycin B Gold (InvivoGen, San Diego, CA, USA).

### 4.2. Mice

Female six-week-old C57BL/6, BALB/c, and athymic BALB/c nude mice were purchased from Orient Bio Inc. (Seongnam, Korea) and maintained in specific pathogen-free conditions. The animal experiments were performed according to the institutional animal care and use committee, approved by the Korea Institute of Radiological & Medical Sciences (KIRAMS 2018-15).

### 4.3. In Vivo Tumor Challenge and Treatment

In the first experimental model, B16F10 cells (1 × 10^6^ cells) were intravenously injected into tail vein with 100 μL phosphate-buffered saline (PBS) after whole-body LDI exposure on C57BL/6 mice. According to previous results [27], the mice were whole-body irradiated with a total dose of 10 cGy delivered in either a single or 10 fractions (1 cGy exposure per day for 10 days) with a ^137^Cs gamma LDI-KCCH 137 irradiator (dose rate 0.1 cGy/min). 

In the second model, 4T1/luc cells were subcutaneously injected into the right thigh (1 × 10^6^ cells/100 μL PBS) of whole-body LDI-treated BALB/c mice. Additionally, LDI-treated 4T1/luc cells were subcutaneously injected into the thighs of the mice (1 × 10^6^ cells/100 μL PBS). Tumor volume was calculated 2–3 times a week after tumor administration using three orthogonal planes (V = (L × W × W)/2, where V is tumor volume, L is tumor length, W is tumor width,). 

In the third tumor model to determine the abscopal response, 4T1 cells were subcutaneously transplanted into the BALB/c back (5 × 10^3^ cells, primary tumor) and the right thigh (1 × 10^3^ cells, secondary tumor) on days 0 and 2, respectively. The mice were randomly assigned to the following groups: control, LDI, XRAD (high-dose RT), and LDI + XRAD. The LDI treatment was administered for 10 consecutive days, starting from day 2. Half of the LDI-treated mice were randomly selected and locally irradiated with high-dose radiation with 8 Gy on days 6, 7, and 8 using the small animal image-guided irradiation system X-RAD SmART (Precision X-ray Inc., North Branford, CT, USA). To deliver accurate doses of radiation to tumors with minimal or no damage to surrounding normal cells, including the immune system, Micro-CT scanning was performed prior to irradiation. A technical description of X-RAD SmART is available at: https://www.accela.eu/precision-x-ray/x-rad-smart. 

### 4.4. Bioluminescence Imaging

To track and compare the metastatic states of the lung using our in vivo models, bioluminescent output from 4T1/luc cells were imaged using IVIS 100 imaging system (Xenogen Corporation, Alameda, CA, USA) after intraperitoneally injection of luciferase substrate 100 mM D-luciferin (Xenolight™ D-Luciferin Potassium salt, PerkinElmer, EU) for 10 min prior to imaging. Mice were anesthetized with vaporized isoflurane (Ifran, Hana Pharm. Co. Ltd. Kyonggi-do, Korea) and placed in imaging chambers. Bioluminescent signal is represented in the images with a pseudo-color scale ranging from red (most intense) to violet (least intense), indicating the intensity of the signal. 

### 4.5. Hematological Analysis

Blood samples were collected via the retroocular plexus bleeding procedure using heparinized capillary tubes from individual mice at the sacrificed times, and complete blood cell counts were obtained using a VETSCAN HM5 hematology analyzer (Abaxis, Union City, CA, USA).

### 4.6. 3-[4,5-Dimethylthiazol-2-yl]-2,5-diphenyltetrazolium bromide (MTT) Assay

Isolated B16F10 cells from metastatic lung tissue were grown in DMEM and subjected to the assay of cellular function after two passages. Cell viability was assessed using the MTT assay (Sigma-Aldrich, St. Louis, MO, USA) and specific procedures were conducted as previously described [67]. All experiments were performed in triplicate.

### 4.7. Wound Scratch Assay

Isolated B16F10 cells were seeded into 6-well plates at 90% confluency and incubated overnight. On the next day, cells were scratched using a 200-μL pipette tip. After wounding, the cells were allowed to migrate for 24 h and subsequently stained with crystal violet. Cell migration was examined under a microscope and photographed at a magnification of × 400.

### 4.8. Western Blot Assay

Total proteins from the isolated cells and tissues were extracted in TNN buffer (50 mM Tris-Cl, pH 7.4, 1% NP-40, 150 mM NaCl, and 1 mM EDTA) supplemented with protease inhibitors (1 mM PMSF, 1 μg/mL aprotinin and 1 μg/mL leupeptin) and phosphatase inhibitors (1 mM Na_3_VO_4_ and 1 mM NaF). Protein samples (15 μg) were separated by SDS-polyacrylamide gel electrophoresis and transferred to nitrocellulose membranes (Bio-Rad, Hercules, California, CA, USA). After blocking non-specific antibody binding sites, the membrane was incubated overnight at 4 °C with a mouse monoclonal antibody against α-SMA (ab15734, Abcam, Cambridge, MA, USA), collagen I (ab138492, Abcam), E-cadherin (sc-8426, Santa Cruz Biotechnology, Paso Robles, CA, USA), fibronectin (sc-8422, Santa Cruz Biotechnology), N-cadherin (sc-393933, Santa Cruz Biotechnology), MMP2 (13132, Cell signaling, Danvers, MA, USA), vimentin (sc-32322, Santa Cruz Biotechnology) and β-actin (A5316, Sigma–Aldrich). All the antibodies were used at a dilution of 1:1,000. After incubation with peroxidase-conjugated secondary antibodies at 37 °C for 1 h, the generated protein bands were visualized using an enhanced chemiluminescence reagent (Cyanagen, Bologna, Italy) and detected using the Amersham Imager 680 (GE Healthcare Biosciences, Little Chalfont, UK). All experiments were performed in triplicate. 

### 4.9. Phenotypic Analysis of Splenocytes

Isolated splenocytes (1 × 10^6^ cells/tube) from individual mice were stained with the following monoclonal antibodies purchased from BD Biosciences (San Diego, CA, USA) and analyzed according to the method reported in a previous study [24]: APC hamster anti-TCRβ (H57-597), APC rat anti-CD4 (RM4-5), APC rat anti-CD8 (53-6.7), APC rat anti-B220 (RA3-6B2), FITC mouse anti-NK1.1 (PK136), APC hamster anti-CD11c (HL3), Alexa Flour® 647 hamster anti-CD28 (37.51), APC rat anti-CD25 (PC61), FITC rat anti-CD44 (IM7), FITC rat anti-CD19 (1D3), FITC hamster anti-CD69 (H1.2F3), FITC hamster anti-CD80 (16-10A1), and FITC rat anti-CD86 (GL1), and eBioscience (San Diego, CA, USA): FITC rat anti-F4/80 (BM8) and APC rat anti-CD11b (M1/70). 

### 4.10. Immunofluorescence Staining

Lung and tumor tissues from experimental mice were harvested and embedded in OCT compound (Tissue-Tek, Torrance, CA). Cryosections (4-5 μm) were fixed in 100% acetone, followed by blocking with 3% bovine serum albumin (GenDEPOT, Barker, TX, USA) solution. The sections were stained with anti-CD3 (ab16669, Abcam), anti-CD4 (NBP1-19371, Novus Biologicals, CO, USA), anti-CD8 (ab4055, Abcam), anti-CD11c (ab11029, Abcam), anti-F4/80 (ab6640, Abcam), fibronectin (ab2413, Abcam) and vimentin (ab193555, Abcam) overnight at 4 °C. On the next day, sections were incubated with an Alexa Fluor^®^ 488- and 647-labeled secondary antibody (Abcam). Images of issues were acquired using a LSM880 confocal microscope (Zeiss, Jena, Germany) using the Zen 2.3 software (Zeiss) for image processing and quantitative analysis. The arithmetic mean of the optical intensities of the target proteins were measured in individual slide and normalized to those of DAPI (4′,6-diamidino-2-phenylindole) using the Zen 2.3 software (Zeiss, Jena, Germany). All experiments were performed in three randomly selected groups.

### 4.11. Hematoxylin and Eosin Staining

Lung tissues were stained with Harris’ hematoxylin (Thermo scientific, Waltham, MA, USA) and eosin Y ethanol solution (Sigma-Aldrich), according to the manufacturer’s instructions. The stained tissues were analyzed using an Axio Imager M2 (Zeiss) microscope.

### 4.12. Statistical Analysis

Statistical analyses were performed using GraphPad software version 5 (GraphPad, La Jolla, CA, USA). All data are expressed as means ± standard errors of means. Significant differences between the groups were determined by analysis of variance and Tukey’s post hoc test. *p* values lower than 0.05 were considered statistically significant, as indicated in figure legends.

## 5. Conclusions

Despite the increasing data highlighting the biological effect of LDI and the growing interest in the use of LDI in clinical practice, preclinical study reports are rare. Therefore, the aim of this study was to evaluate anti-tumor effects of whole-body LDI in metastatic mouse models. The pretreatment of fractionated LDI in mice significantly inhibited lung metastasis through the suppression of EMT-related proteins and infiltration of immune cells. The reduction in tumor motility and invasive function by LDI resulted in successful inhibition of micrometastases in lung. The combination of high-dose RT and LDI significantly delayed both primary and secondary tumor growth. As metastasis is responsible for cancer-related death and poor prognosis, this approach may help to present a novel, cost-effective strategy for treating metastasis compared to with combination therapy of RT and IT. To do this, challenges to clarify underlying mechanisms of LDI as an immune-booster and a modulator of TME are needed. In summary, this study demonstrates that LDI may play an important role in boosting the high-dose RT-mediated abscopal effect by preventing the EMT process and inducing systemic immune responses. 

## Supplementary Materials

The following are available online at https://www.mdpi.com/2072-6694/12/5/1126/s1, Figure S1: Detailed information about western blot of Figure 1E, Figure S2: Detailed information about western blot of Figure 4A, Figure S3: Reduction of metastatic potentials of LDI-treated 4T1 cells.
