# Supplementary material for: Evaluation of Anti-Tumor Effects of Whole-Body Low-Dose Irradiation in Metastatic Mouse Models
Source: Cancers (Basel). 2020 Apr 30;12(5):1126. doi: 10.3390/cancers12051126 (PMC7281283; doi:10.3390/cancers12051126)
Supplement: Supplementary file 1 [file cancers-12-01126-s001.pdf]

Supplementary Materials

Evaluation of Anti-Tumor Effects of Whole-Body Low-Dose Irradiation in Metastatic Mouse Models

Kyung-Hee Song, Seung-Youn Jung, Jeong-In Park, Jiyeon Ahn, Jong-Kuk Park, Sang-Gu Hwang, Eun-Ho Kim, Seon Young Nam, Seungwoo Park, Hunjoo Ha and Jie-Young Song

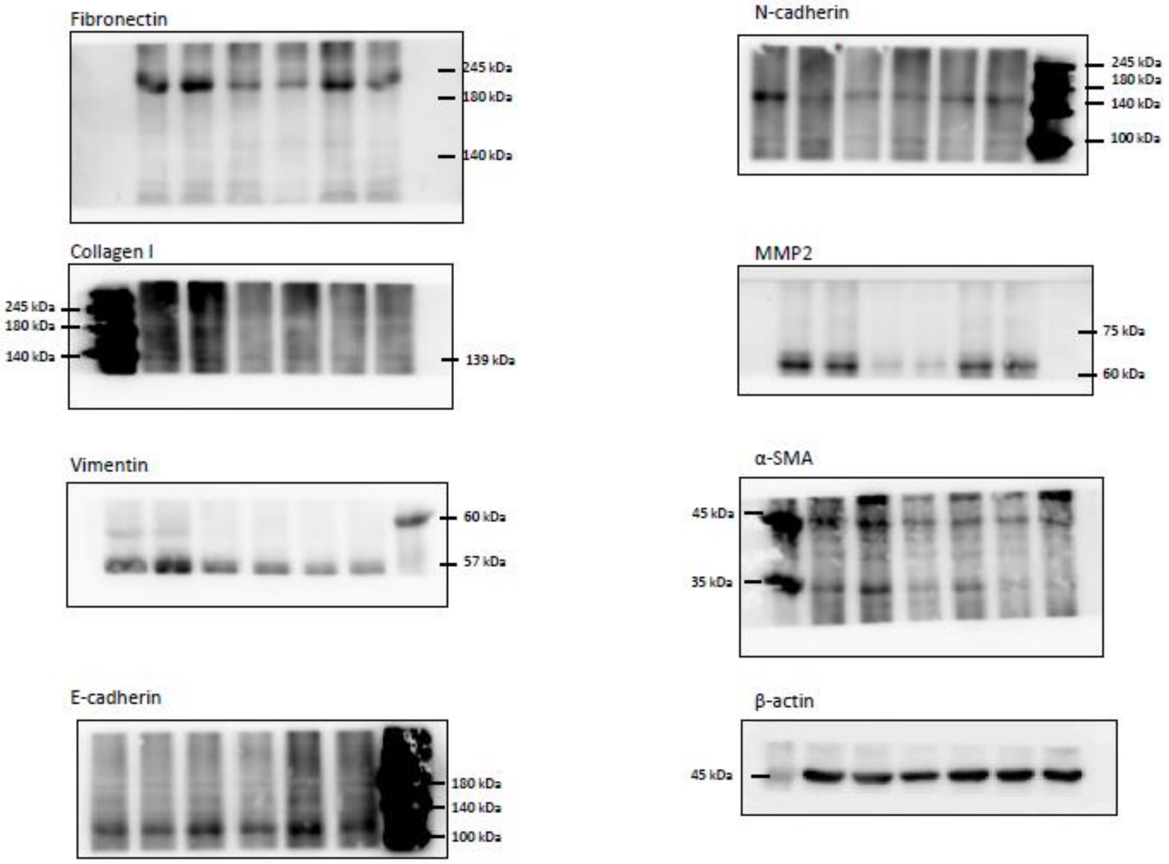

Figure S1. Detailed information about western blot of Figure 1E.

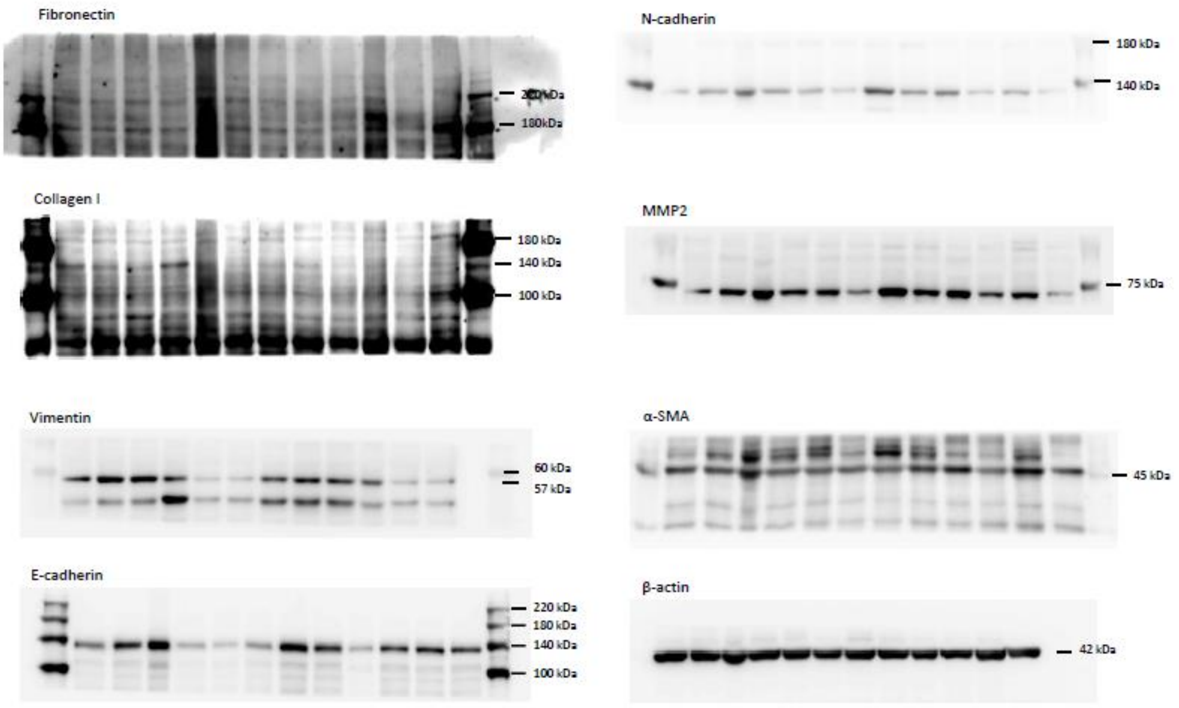

Figure S2. Detailed information about western blot of Figure 4A.

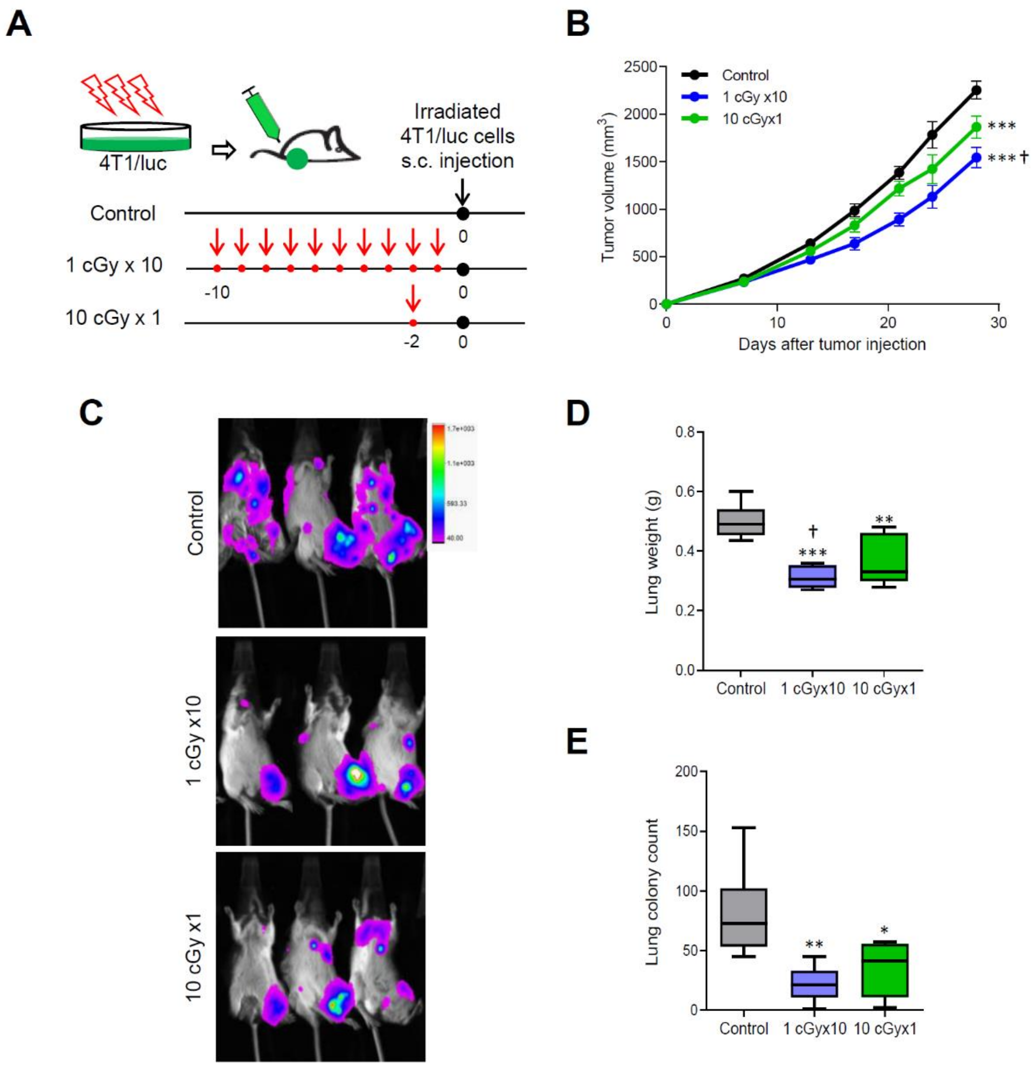

Figure S3. Reduction of metastatic potentials of LDI-treated 4T1 cells. (A) Experimental scheme of directly LDI exposure to 4T1/luc cells. 4T1/luc cells were either irradiated with 10 cGy at once or 1 cGy for 10 times (accumulative dose of 10 cGy), and then were s.c. injected into right thigh. (B) Tumor growth was measured after LDI-treated 4T1/luc transplantation. (C) Bioluminescent intensity of representative mice was monitored on week 34 after injection of 4T1/luc cells. (D) Lung weights. (E) The number of metastatic foci were counted and calculated in each group. Data represent means ± SEM of 6-7 mice experiment. * $p < 0.05$, ** $p < 0.01$, *** $p < 0.001$ vs. control; † $p < 0.05$ vs. 10 cGy x 1.

© 2020 by the authors. Licensee MDPI, Basel, Switzerland. This article is an open access article distributed under the terms and conditions of the Creative Commons Attribution (CC BY) license (http://creativecommons.org/licenses/by/4.0/).
